# Targeting Membrane Lipid a Potential Cancer Cure?

**DOI:** 10.3389/fphar.2017.00012

**Published:** 2017-01-23

**Authors:** Loh Teng-Hern Tan, Kok-Gan Chan, Priyia Pusparajah, Wai-Leng Lee, Lay-Hong Chuah, Tahir Mehmood Khan, Learn-Han Lee, Bey-Hing Goh

**Affiliations:** ^1^Novel Bacteria and Drug Discovery Research Group, School of Pharmacy, Monash University MalaysiaBandar Sunway, Malaysia; ^2^Division of Genetics and Molecular Biology, Institute of Biological Sciences, Faculty of Science, University of MalayaKuala Lumpur, Malaysia; ^3^Biomedical Research Laboratory, Jeffrey Cheah School of Medicine and Health Sciences, Monash University MalaysiaBandar Sunway, Malaysia; ^4^School of Science, Monash University MalaysiaSelangor, Malaysia; ^5^Department of Pharmacy, Abasyn University PeshawarPeshawar, Pakistan; ^6^Center of Health Outcomes Research and Therapeutic Safety, School of Pharmaceutical Sciences, University of PhayaoPhayao, Thailand

**Keywords:** phosphatidylethanolamine, phospholipid bilayer, targeted drug, anticancer, membrane permeabilization

## Abstract

Cancer mortality and morbidity is projected to increase significantly over the next few decades. Current chemotherapeutic strategies have significant limitations, and there is great interest in seeking novel therapies which are capable of specifically targeting cancer cells. Given that fundamental differences exist between the cellular membranes of healthy cells and tumor cells, novel therapies based on targeting membrane lipids in cancer cells is a promising approach that deserves attention in the field of anticancer drug development. Phosphatidylethanolamine (PE), a lipid membrane component which exists only in the inner leaflet of cell membrane under normal circumstances, has increased surface representation on the outer membrane of tumor cells with disrupted membrane asymmetry. PE thus represents a potential chemotherapeutic target as the higher exposure of PE on the membrane surface of cancer cells. This feature as well as a high degree of expression of PE on endothelial cells in tumor vasculature, makes PE an attractive molecular target for future cancer interventions. There have already been several small molecules and membrane-active peptides identified which bind specifically to the PE molecules on the cancer cell membrane, subsequently inducing membrane disruption leading to cell lysis. This approach opens up a new front in the battle against cancer, and is of particular interest as it may be a strategy that may be prove effective against tumors that respond poorly to current chemotherapeutic agents. We aim to highlight the evidence suggesting that PE is a strong candidate to be explored as a potential molecular target for membrane targeted novel anticancer therapy.

## Introduction

Cancer represents a major health concern globally. In 2012, cancer was responsible for 8 million deaths, with an estimated 14 million new cases diagnosed. Recent decades have seen advances in the diagnosis and treatment of cancer, but the overall reduction in cancer mortality is still limited; and even more worryingly, cancer-related cases and deaths are projected to rise by at least 70% by 2030 (Ferlay et al., 2015; Antoni et al., 2016). Even if patients do successfully overcome cancer through the current standard mainstays of treatment —namely chemotherapy and radiotherapy— the risk of reoccurrence of the disease is a major concern. Currently, the main issues of concern relating to the currently available therapeutic options are low therapeutic indices and a broad spectrum of adverse effects (MacDonald, 2009). The key issues underlying problems of potential toxicity as well as drug resistance are attributed to the lack of specificity of existing therapies toward tumor cells (Chari, 2007). Drug resistance can be intrinsic because of variations between individual patients and also due to the genetic differences in tumors (Gottesman et al., 2002). Increasing evidence has demonstrated various mechanisms by which cancer cells may acquire resistance such as drug-detoxifying mechanisms, expression of one or more energy-dependent transporters that extrude anticancer agent from the cells before interaction with intracellular targets take place, or acquiring the ability to evade drug induced apoptosis (Gottesman et al., 2002; Perez-Tomas, 2006; Gillet and Gottesman, 2010; Batist et al., 2011). Taken together, there is a mandate to develop innovative therapeutic strategies to overcome the limitations of current therapy, subsequently lowering the ever-increasing cancer related mortality and morbidity.

In the late 1800s, bacteriologist Paul Ehrlich first conceptualized the ‘magic bullet,’ a specifically synthesized drug that would target a specific microorganism while not harming any other cells in the organism. This concept has now evolved into a more general field aiming to develop targeted drug delivery strategies (Imai and Takaoka, 2006) to treat various diseases including cancer and inflammatory diseases (Allen and Cullis, 2004). Currently, these efforts have resulted in the development of a full range of powerful molecular therapeutics including monocompounds with targeting properties or a cocktail of therapeutic compounds which may comprise fusion proteins and nano/microparticles. The clinical success of targeted drug delivery is highly dependent on its delivery vehicle and its targets. In cancer treatment, a number of targeting functions have been discovered such as tumor-antigen recognizing antibodies (Kirpotin et al., 2006), ligands for receptors (Pastorino et al., 2006) and RNA aptamers (Farokhzad et al., 2006) against antigens which are expressed on the surface of tumor cells. However, actually utilizing this discovery in developing targeted drug delivery systems is limited by virtue of the lack of an ubiquitously expressed tumor-specific antigen or receptor on various cancer cells. Also, the geno- and phenotypic heterogeneity of individual tumors (Marusyk et al., 2012) and the co-existence of drug-susceptible and drug-resistant clones (Greaves and Maley, 2012) further complicates targeted therapy.

**FIGURE 1:**
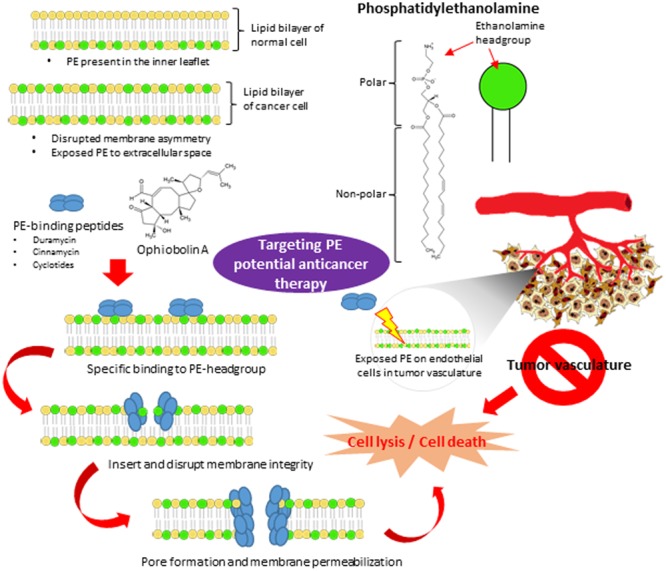
**The potential of PE as the novel molecular therapeutic target in anticancer treatment.** The plasma membrane of normal cells is characterized by an asymmetric distribution of various phospholipids over two membrane leaflet. PE resides in the inner leaflet facing the cytosol. The disrupted membrane asymmetry of cancer cell with exposed PE to extracellular space serves as a molecular target for anticancer therapy. PE-binding peptides and small molecules (ophiobolin A) bind specifically to the ethanolamine headgroup of PE. Upon binding, they disrupt and induce membrane destabilization via insertion into the lipid bilayer. They also may induce membrane permeabilization via pore formation, subsequently lead to cell lysis and cell death. Furthermore, PE is also highly exposed on endothelium cells in tumor vasculature. Targeting PE on endothelial cells in tumor vasculature could be a potential anti-angiogenesis approach to inhibit tumor development.

In addition to the concerns involving treatment of localized cancers, to date there has also been a lack of success in developing an effective treatment for disseminated cancer. Over the last few decades, it has been shown that the overall contribution of curative and adjuvant cytotoxic chemotherapy has not resulted in substantial improvement of treatment outcomes for most cancer types (Bailar and Gornik, 1997; Morgan et al., 2004; Ashdown et al., 2015). Thus, there is a clearly an urgent need to pursue ground breaking, innovative ideas in developing alternative chemotherapeutics that act specifically on clearly defined biomarkers which are generally expressed within certain tumor types in order to improve cancer cell selectivity of chemotherapeutic agents. Considerable effort has been devoted to developing novel targeted anticancer strategies such as specific agents able to inhibit cancer cell proliferation (Parenti et al., 2014), promote cell cycle regulation (Gabrielli et al., 2012) and induce apoptosis or autophagy (Amaravadi et al., 2011). However, a promising new approach targeting the cancer cell membrane seems to have been underexplored thus far. This approach focuses on destroying cancer cells by damaging their cell membranes instead of binding to specific receptors. Here we aim to highlight the prospect of phosphatidylethanolamine (PE), an aminophospholipid on the cell membrane, as an attractive target for anti-cancer therapy. A schematic diagram depicting the potential of PE as molecular target for peptides and small molecules exhibiting anticancer properties (**Figure 1**) has been included.

## Membrane Targeted Therapy

Generally, most marketed drugs elicit their effects via direct interaction with proteins (Melnikova and Golden, 2004; Adrian et al., 2007); in fact, many new molecular entities are developed for clinical use based on the previously elucidated structure of their intended target proteins (Prathipati et al., 2007). However, as increasing evidence emerges regarding the role of membrane lipids in the regulation of numerous cellular functions and activity of membrane proteins, membrane lipids have emerged as an alternative molecular target (Lee, 2004, 2011). Given that many cellular functions occur in or around the cell membrane, alterations in the types and/or levels of membrane lipids may have implications for many human pathologies, hence it was proposed that therapies based on the regulation of membrane lipids structure could be developed to control the molecular events that are relevant to the various pathological states (Escribá, 2006). This has led to membrane-lipid therapy, which aims to reverse the pathological state of the cell membrane by using drugs that influence the membrane’s lipid organization via the principle of structure-function, subsequently inducing changes in cell signaling and gene expression (Escribá, 2006).

The development of cell membrane targeted cancer therapy is made possible by the fact that there are fundamental differences between the cell membrane composition of normal and cancer cells. Non-cancer cells usually exhibit a more neutral total membrane charge attributed to the presence of zwitterionic phospholipids such as phosphatidylcholine and sphingomyelin while phosphatidylserine (PS) and PE (are) located in the inner leaflet of plasma membrane (Bevers et al., 1996). The asymmetrical distribution of the phospholipids is maintained by a group of P-type ATPases, aminophospholipid translocases that use ATP hydrolysis to flip PS and PE from the external to cytosolic leaflet (Chen et al., 1999). Unlike normal cells, apoptotic cells and cancer cells lose their capacity to maintain PS asymmetry, leading to the exposure of this lipid at the cell’s outer membrane leaflet. PS is one of the molecules that has gained interest in various clinical disciplines, particularly in the development of non-invasive imaging technology to support diagnosis and evaluation of treatment efficacy for various diseases such as cancer and cardiovascular disease (Kenis and Reutelingsperger, 2009). Furthermore, PS also represents an attractive target for targeted drug delivery strategies that aim to treat disease effectively with minimal detrimental side-effects. Due to the limited PE-specific probes available to facilitate the study of behavior of cell surface PE in living systems, the functional significance of PE is less well-documented as compared to that of PS. To date, duramycin and cinnamycin are the only two well-known PE-binding peptides that have assisted in unraveling many important biological functions of PE (Zhao, 2011).

## Phosphatidylethanolamine As An Anti-Cancer Target

Phosphatidylethanolamine is the second most abundant phospholipid on mammalian cellular membranes, accounting for approximately 20% of the total phospholipids (Spector and Yorek, 1985). Generally, PE is found predominantly in the inner leaflet of the plasma membrane of viable mammalian cells. In addition, PE is also abundant in the inner membrane of mitochondria (Vance, 2008). Besides functioning as a membrane structural element, the evidence suggests that PE participates in many important pathophysiological cellular processes (Vance, 2008; Calzada et al., 2016). It has been demonstrated that translocation and redistribution of PE occurs during a number of distinct biological events: (1) cell division where it is externalized at the cleavage furrow (Emoto et al., 1996); (2) cell death (for which exposure of PE to extracellular environment is a molecular marker) (Emoto et al., 1997); (3) anticoagulant mechanism where PE acts as a cofactor for protein C (Tian et al., 2006). Given the diversity in its biological roles, PE represents a distinct molecular target among aminophospholipids and other membrane phospholipid components.

The development of PE-specific probes derived from duramycin and cinnamycin has made possible the recent discoveries of many biological functions of PE in living systems. Duramycin is a highly specific PE-binding peptide produced by *Streptoverticillium cinnamoneus* and is closely related to cinnamycin (Ro09-0198) which is produced by *Streptomyces* sp. (Iwamoto et al., 2007). Both duramycin and cinnamycin are lantibiotics, bacteriocins which are characterized by the presence of a high proportion of unusual thioether amino acids (lanthionine and methyllanthionine). Lantibiotics are toxic toward many Gram-positive bacteria, but the toxic effects of cinnamycin are not limited to bacterial cells. Duramycin and cinnamycin were also found to display a number of effects on eukaryotic cells, such as induction of hemolysis (Choung et al., 1988a), inhibition of phospholipase A2 or interference with prostaglandin and leukotriene biosynthesis (Märki et al., 1991). Most of these effects described can be explained by the specific binding of these lantibiotics to PE (Choung et al., 1988b; Hosoda et al., 1996).

Both duramycin and cinnamycin are unique in that they bind to PE located in the inner layer of the plasma membrane in mammalian cells. They bind with high affinity to the head group of PE at a molar ratio of 1:1 with a dissociation constant in the nanomolar range (Zhao et al., 2008). Given that they specifically bind to PE, they have been employed to study the distribution and metabolism of PE in different biological systems. The binding of cinnamycin to PE was found to induce transbilayer phospholipid movement of target cells in a PE-dependent manner (Makino et al., 2003). This study demonstrated that the binding of cinnamycin induces membrane reorganization that eventually leads to cell death (Makino et al., 2003). In addition to inducing transbilayer lipid movement, there is preferential binding of both duramycin and cinnamycin to areas of membrane with high curvature and further binding of the peptides is promoted via membrane tubulation (Iwamoto et al., 2007).

In addition to duramycin and cinnamycin, cyclotides represent a new lipid-binding protein family of cyclic peptides which exert their biological activities by acting on cell membrane, particularly by binding to phospholipids containing PE headgroups. Studies have shown that the cyclotides interact and bind specifically with membranes containing PE-phospholipids as compared to other lipids lacking PE-phospholipids and their binding was followed by insertion that subsequently led to membrane disruption (Henriques et al., 2011, 2012; Troeira Henriques et al., 2014). Wang et al. (2012) proposed that the binding of cyclotides to cell membrane surface via interaction with PE-lipid headgroup resulted in formation of pores based on barrel-stave or toroidal pore models when a threshold concentration is exceeded. The toxicity of cyclotides were shown to be dependent on PE exposure at the membrane surface. Although cyclotides were found to be equally toxic toward cancerous and non-cancerous cell, cyclotides can recognize cancer cells more efficiently than non-cancerous cells at non-permeabilising concentrations (Troeira Henriques et al., 2014). Hence, the higher proportion of exposed PE on the outer membrane of cancer cell presents a potential molecular target for those membrane-active peptides to exert their cytotoxic effects. In addition, PE was found to play a role in enhancing the susceptibility of the membrane to permeabilization by bound peptides and also facilitating the formation of larger transmembrane pores (Leite et al., 2015). These findings open up new avenues toward the development of novel chemotherapeutic peptides that specifically recognize PE phospholipid components on cancer cells and induce membrane disruption upon binding, leading to membrane permeabilization via pore formation and eventually cell death.

Despite numerous successful examples of membrane active peptides in *in vivo* studies, to date none of them have made it into the pharmaceutical market. Potential issues including poor bioavailability and potential toxicity of peptides are hampering the progress and development of peptides as novel anticancer agents. Furthermore, peptide degradation due to serum proteases is also a major challenge limiting the stability and half-life of these molecules (Adessi and Soto, 2002).

A recent study has found that a natural product, ophiobolin A (OPA) exerts its cytotoxicity toward cancer cells by forming a pyrrole-containing covalent cytotoxic adduct with the ethanolamine head group of PE, subsequently leading to lipid bilayer destabilization (Chidley et al., 2016). OPA is a plant toxin isolated from pathogenic fungi of the *Bipolaris* genus that exhibits cytotoxicity at nanomolar concentrations against a range of cancer cell lines. Basically, the study performed an unbiased genome-wide approach to identify the molecular target of OPA through insertional mutagenesis of near-haploid cell line KBM7 using retroviral gene trap approach and demonstrated that OPA forms PE-OPA adducts associated with membrane disruption and eventual cell death (Chidley et al., 2016). This discovery highlighted the potential importance of PE as the molecular target for small molecules which provides further impetus to pursue targeting PE as a novel chemotherapeutic approach, particularly against cancer types which are known to respond poorly to current chemotherapy.

Additional information from a recent study revealed that PE is highly exposed on tumor vascular endothelium, suggesting that it may serve as a marker for imaging tumor vasculature and also drug targeting (Stafford and Thorpe, 2011). Targeting the angiogenesis-driven sprouting of new vessels has seen a revolution in anti-cancer drug development in the past decade. Angiogenesis and tumor vascularization are crucial to the growth and metastasis of cancers (Folkman, 2002; Berretta et al., 2016). The observation that tumors cannot grow beyond a size of approximately 2 mm^3^ without the support of neovascularization (Folkman, 1971) has led to clinical development of a large number of angiogenesis-inhibiting agents that act on a number of potential targets, including the matrix-metalloproteinases, tissue plasminogen activator (Pepper, 2001), angiogenic cytokines and vascular endothelial cells (Dvorak, 2002) participating in angiogenesis. Therefore, the selective exposure of PE on tumor vasculature may present an opportunity for selective therapeutic intervention, whereby specific PE-binding peptides or antibodies can be employed to direct or deliver the bound endothelial cell-damaging agents to the site of action.

## Conclusion and Future Directions

To date, the success of cancer therapies is limited by their severe side effects and also the problems of drug resistance. These issues make it vital to explore potential molecular targets for anticancer therapy like PE which have strong potential for translation into future clinical applications. A number of membrane-active peptides and small molecules have been shown to bind PE specifically, subsequently eliciting membrane disruption. The ability of those molecules to recognize cancer cells is PE dependent, suggesting that PE is an efficient means of targeting cancers. This notion is strengthened further by the finding that the levels of PE exposure on the outer membrane leaflet is particularly high on cell surfaces exposed to stress conditions associated with tumor microenvironments. However, optimization and modification of peptides are required to enhance their selective interaction with their target on the cell membrane as well as reducing toxicity and development of drug resistance. The knowledge of these PE-binding peptides and small molecules has the potential to assist in the design of novel drugs based on their molecular scaffold, perhaps allowing development of molecules capable of targeting specific cell types. Based on the concept of membrane disruption via binding of PE, suitable assays employing high-throughput screening strategy can be used to identify novel PE-binding molecules from large libraries of drug candidates before optimization in terms of their potency, selectivity, physicochemical, pharmacokinetic, and toxicity properties.

## Author Contributions

LT contributed to the literature database search and writing of the manuscript. L-HL, K-GC, W-LL, L-HC, TK, PP, and B-HG contributed vital insights and proofread the writing. The research topic was conceptualized by B-HG.

## Conflict of Interest Statement

The authors declare that the research was conducted in the absence of any commercial or financial relationships that could be construed as a potential conflict of interest.

The reviewer CP and handling Editor declared their shared affiliation, and the handling Editor states that the process nevertheless met the standards of a fair and objective review.
